# Improving mortality data in Jordan: a 10 year review

**DOI:** 10.2471/BLT.14.137190

**Published:** 2015-08-14

**Authors:** Faris Dababneh, Erin K Nichols, Majed Asad, Yousef Haddad, Francis Notzon, Robert Anderson

**Affiliations:** a Directorate of Information and Research, Ministry of Health, Amman, Jordan.; b National Center for Health Statistics, Centers for Disease Control and Prevention, United States Public Health Service, Hyattsville, United States of America (USA).

## Abstract

**Problem:**

Before 2003 there was substantial underreporting of deaths in Jordan. The death notification form did not comply with World Health Organization (WHO) guidelines and information on the cause of death was often missing, incomplete or inaccurate.

**Approach:**

A new mortality surveillance system to determine the causes of death was implemented in 2003 and a unit for coding causes of death was established at the ministry of health.

**Local setting:**

Jordan is a middle-income country with a population of 6.4 million people. Approximately 20 000 deaths were registered per year between 2005 and 2011.

**Relevant changes:**

In 2001, the ministry of health organized the first meeting on Jordan’s mortality system, which yielded a five-point plan to improve mortality statistics. Using the recommendations produced from this meeting, in 2003 the ministry of health initiated a mortality statistics improvement project in collaboration with international partners. Jordan has continued to improve its mortality reporting system, with annual reporting since 2004. Reports are based on more than 70% of reported deaths. The quality of cause-of-death information has improved, with only about 6% of deaths allocated to symptoms and ill-defined conditions – a substantial decrease from the percentage before 2001 (40%). Mortality information is now submitted to WHO following international standards.

**Lessons learnt:**

After 10 years of mortality surveillance in Jordan, the reporting has improved and the information has been used by various health programmes throughout Jordan.

## Résumé

**Problème:**

Avant 2003, le nombre de décès était considérablement sous-déclaré en Jordanie. Le formulaire de déclaration de décès n'était pas conforme aux directives de l'Organisation mondiale de la Santé (OMS) et les informations relatives aux causes des décès étaient généralement manquantes, incomplètes ou imprécises.

**Approche:**

Un nouveau système de surveillance de la mortalité pour déterminer les causes de décès a été mis en place en 2003 et une unité chargée de codifier les causes de décès a été créée au sein du ministère de la Santé.

**Environnement local:**

La Jordanie est un pays à revenu intermédiaire qui compte 6,4 millions d'habitants. Environ 20 000 décès par an ont été enregistrés entre 2005 et 2011.

**Changements significatifs:**

En 2001, le ministère de la Santé a organisé une première réunion sur le système de surveillance de la mortalité de Jordanie, qui a conduit à l'élaboration d'un plan en cinq points visant à améliorer les statistiques de mortalité. À partir des recommandations formulées au cours de cette réunion, le ministère de la Santé a amorcé en 2003 un projet d'amélioration des statistiques de mortalité en collaboration avec des partenaires internationaux. La Jordanie a poursuivi l'amélioration de son système d'établissement de rapports sur la mortalité avec l'élaboration depuis 2004 de rapports annuels. Ces rapports s'appuient sur plus de 70% des décès signalés. La qualité des informations relatives aux causes des décès s'est améliorée, seulement 6% environ des décès étant attribués à des symptômes et à des troubles mal définis, ce qui représente une baisse importante par rapport au pourcentage antérieur à 2001 (40%). Les données de mortalité sont désormais soumises à l'OMS conformément aux normes internationales.

**Leçons tirées:**

Après 10 ans de surveillance de la mortalité en Jordanie, la diffusion de données s'est améliorée et les informations ont été utilisées dans le cadre de différents programmes de santé dans l'ensemble du pays.

## Abstract (es)

**Situación:**

Antes de 2003, existía una infradeclaración sustancial de las muertes en Jordania. El formulario de notificación de defunciones no cumplía con las directrices de la Organización Mundial de la Salud (OMS), ya que en repetidas ocasiones la información referente a la causa del fallecimiento no se proveía, o bien estaba incompleta o era incorrecta.

**Enfoque:**

En 2003, se implementó un nuevo sistema de vigilancia de la mortalidad para determinar las causas de mortalidad. También se estableció una unidad para clasificar las causas de mortalidad en el Ministerio de Salud.

**Marco regional:**

Jordania es un país de ingresos medios y tiene una población de 6,4 millones de personas. Entre 2005 y 2011, se registraron unas 20.000 defunciones por año.

**Cambios importantes:**

En 2001, el Ministerio de Salud organizó la primera reunión sobre el sistema de mortalidad de Jordania, cuyo resultado fue un plan de cinco puntos para mejorar las estadísticas de mortalidad. Haciendo uso de las recomendaciones que salieron de esa reunión, en 2003, el Ministerio de Salud inició un proyecto de mejora de las estadísticas de mortalidad en colaboración con socios internacionales. Jordania ha seguido mejorando su sistema de presentación de informes sobre mortalidad, con informes anuales desde 2004. Dichos informes se basan en más del 70% de las defunciones notificadas. La calidad de la información referente a la causa de mortalidad ha mejorado, con solo un 6% de las defunciones atribuidas a síntomas y estados no definidos, la cual cosa significa una caída sustancial del porcentaje existente antes de 2001 (40%). Actualmente, la información sobre mortalidad se presenta ante la OMS y cumple con las normas internacionales.

**Lecciones aprendidas:**

Después de 10 años de vigilancia de la mortalidad en Jordania, los informes han mejorado y la información se ha usado para diferentes programas sanitarios por toda Jordania.

## ملخص

**المشكلة:**

شهدت الأردن نقصًا كبيرًا في الإبلاغ عن حالات الوفاة فيما قبل عام 2003. فلم يتوافق نموذج الإخطار بالوفاة مع المبادئ التوجيهية لمنظمة الصحة العالمية (WHO)، كما كانت المعلومات المتعلقة بسبب الوفاة غير موجودة أو غير مكتملة أو غير دقيقة في أغلب الأحيان.

**الأسلوب:**

تم تطبيق نظام جديد في عام 2003 لمراقبة الوفيات بغرض تحديد أسباب الوفاة، كما تم إنشاء وحدة في وزارة الصحة لترميز أسباب الوفاة.

**المواقع المحلية:**

تمثل الأردن دولة متوسطة الدخل يبلغ عدد سكانها 6.4 مليون شخص. وقد تم تسجيل ما يقرب من 20000 حالة وفاة في العام في الفترة ما بين عامي 2005 و2011.

**التغيرات ذات الصلة:**

نظمت وزارة الصحة في عام 2001 أول اجتماع بشأن نظام تسجيل الوفيات في الأردن، وقد تمخض عن خطة مكونة من خمس نقاط لتحسين الإحصائيات المتعلقة بالوفيات. وبناء على التوصيات التي أثمر عنها الاجتماع، بدأت وزارة الصحة في عام 2003 مشروعًا لتحسين الإحصائيات المتعلقة بالوفيات بالتعاون مع شركاء دوليين. واستمرت الأردن في تحسين نظام الإبلاغ عن الوفيات باستخدام الإبلاغ السنوي منذ عام 2004. واستندت التقارير إلى ما يزيد عن 70% من حالات الوفاة المبلغ عنها. وتحسن مستوى جودة المعلومات المتعلقة بأسباب الوفاة، مع وجود حوالي 6% فقط من حالات الوفاة المصنفة ضمن حالات المعاناة من بعض الأعراض المرضية والحالات التي تفتقر إلى دقة التحديد، مما أدى إلى حدوث انخفاض ملموس في النسبة المئوية المسجلة قبل عام 2001 (40%). وتُقدَّم المعلومات المتعلقة بالوفيات حاليًا لمنظمة الصحة العالمية باتباع المعايير الدولية.

**الدروس المستفادة:**

بعد مرور 10 سنوات على تطبيق نظام مراقبة الوفيات في الأردن، تحسّن الإبلاغ عن الوفيات، ويتم استخدام المعلومات المتعلقة بالوفيات بواسطة العديد من البرامج الصحية في مختلف أنحاء الأردن .

## 摘要

**问题:**

在 2003 年之前，约旦漏报了大量死亡人数。 死亡通知表单不符合世界卫生组织 (WHO) 的标准，死亡原因信息经常缺失、不完整或不准确。

**方法:**

一个能判定死亡原因的新型死亡率监测系统在 2003 年得以应用，卫生部还成立了一个给死亡原因编码的单位。

**当地状况:**

约旦是一个有着 640 万人口的中等收入国家。 在 2005 年至 2011 年间，每年登记死亡人数大约有 2 万人。

**相关变化:**

在 2001 年，卫生部组织了关于约旦死亡率监测系统的第一次会议，会议提出了五点计划来提升死亡统计质量。 根据会议提出的建议， 2003 年卫生部与国际伙伴合作发起了提升死亡率统计质量的项目。 约旦不断改进他们的死亡率报告系统，自 2004 年起每年提交一份报告。报告的内容基于超过 70% 的汇报死亡人数。 死亡原因信息的质量已经得到提高，大约只有 6% 的死亡症状情况不明——与 2001 年之前的百分比 (40%) 相比大大减少。 现在，死亡率信息已按照国际标准提交给世界卫生组织。 **经验教训 **约旦经过了 10 年的死亡率监测，报告质量得到提升，信息已被全国各种医疗项目使用。

## Резюме

**Проблема:**

До 2003 года в Иордании имели место значительные проблемы с отчетностью о смертности. Форма извещения о смерти не соответствовала нормам Всемирной организации здравоохранения (ВОЗ), и часто информация о причине смерти отсутствовала, была неполной или неточной.

**Подход:**

В 2003 году была внедрена новая система контроля смертности для определения причин смертей. В Министерстве здравоохранения был создан отдел для кодирования причин смертности.

**Местные условия:**

Иордания — страна с населением 6,4 млн человек со средним уровнем доходов. В период между 2005 и 2011 годами в среднем в год в стране регистрировалось около 20 000 смертей.

**Осуществленные перемены:**

В 2001 году Министерство здравоохранения организовало первое совещание по системе учета смертности в Иордании, результатом которого стал план из пяти пунктов, направленный на улучшение качества статистических данных по смертности. Используя рекомендации, выработанные на этом совещании, Министерство здравоохранения в 2003 году начало проект по улучшению качества статистического учета смертности, для чего были привлечены иностранные партнеры. Иордания продолжила улучшать систему учета смертности, введя с 2004 года ежегодную отчетность. Отчеты основаны более чем на 70% зафиксированных смертей. Как следствие, качество информации о причинах смерти улучшилось. Только 6% смертей описываются как связанные с симптомами и плохо определимыми заболеваниями. Это значительно меньше показателей, наблюдавшихся до 2001 года (40%). В настоящее время данные по смертности, представляемые в ВОЗ, соответствуют международным стандартам.

**Выводы:**

После 10 лет контроля смертности в Иордании отчетность улучшилась, а получаемая информация использовалась в различных программах здравоохранения, проводимых по всей Иордании.

## Problem

As in many other middle-income countries, Jordanian law mandates that all deaths be registered. However, before 2003 registration was not universal and cause of death certification was often inaccurate. A survey conducted in 1995–1996 used verbal autopsy to estimate mortality indicators and determine causes of death.^1^ The death notification form did not comply with World Health Organization (WHO) recommendations^2^ for mortality reporting and data were inconsistent and not comparable within Jordan or with other countries.

Assessing death notification forms from 1996 to 2000, the disease control directorate of the Ministry of Health of Jordan found underreporting of deaths, particularly infant deaths, missing information on cause of death and incomplete and inaccurate reporting of the cause of death. The crude death rate based on registered deaths was about half that estimated by the Department of Statistics (2.4–2.8 versus 5.0 per 1000 population). Over 12% of reports were missing direct cause of death, and 40% of all death notifications contained uninformative causes of death, including symptoms or ill-defined conditions. Before 2003, no cause-of-death coding was completed, inhibiting production of useful mortality statistics. Lack of coding also meant that underlying cause-of-death information could not be provided to WHO in the requested format.

## Approach

The improvement of mortality data in Jordan was initiated by an inter-agency collaboration and led by a mortality surveillance unit. In March 2001, Jordan enacted a new law requiring deaths to be reported and a burial permit sought within 10 days of death. In December 2001, the ministry of health organized the first national meeting on the Jordanian mortality system with WHO, the United States Centers for Disease Control and Prevention (CDC), and the United States Agency for International Development (USAID). Approximately 40 people attended, including health sector officials, medical and paramedical syndicates, deans of medical schools, mortality statistics experts, staff from the Department of Statistics and the Civil Status and Passports Department (which is directly responsible for death notification in Jordan). The multi-agency task force proposed a five-point plan to improve mortality statistics in Jordan: (i) establishment of a cause-of-death coding unit at the ministry of health, Directorate of Information and Research; (ii) modification of death notification form; (iii) training on use of the WHO International Classification of Diseases and Related Health Problems (ICD) for cause-of-death certification and coding; (iv) appointment of focal points for supervision and quality control; and (v) tabulation and reporting.

Following these recommendations, the ministry of health initiated a project in 2003 to improve mortality statistics in collaboration with WHO, CDC and USAID. The project included a new mortality surveillance system to monitor causes of death and a unit for coding causes of death.

### Modified notification form

The death notification form was modified to comply with WHO recommendations. The modified form now includes a two part medical certification section, four lines for recording cause of death and space for recording the period between onset of cause and death. These changes facilitate accurate cause-of-death certification and the application of WHO recommendations^2^ for selecting the underlying cause of death. The attending physician completes the notification in duplicate, with the original sent to the Civil Status and Passports Department and the duplicate copy sent to the ministry of health. The modified form was distributed throughout Jordan in the last six months of 2003.

### Certification and coding

The ministry of health developed a one-page guideline on completing the modified death notification form and distributed it to all physicians and hospitals. A telephone service was established during working hours for enquiries. Workshops were held in the largest ministry of health hospitals, and orientation workshops were held for new employees. In 2003 the ministry of health sent 10 staff physicians to be trained as mortality coders by the Australian National Centre for Classification in Health. The training covered all chapters of the ICD (10th revision) manual coding procedures including four-digit coding and medical terminology. Two physicians were assigned to the mortality coding group in the ministry of health coding unit and Jordan began officially using the ICD system for mortality coding for 2004 data. With support from WHO, the coding unit conducts a workshop on how to use ICD-10 every two years.

### Supervision and quality control

Training as focal points for completing the death notification form was provided for 50 staff from 20 health directorates and 30 public hospitals, five forensic medicine staff and one staff member from each of the Royal Medical Services, Jordan University Hospital and Jordan University for Science and Technology. The focal points were appointed in their respective organizations to train colleagues, review forms for accuracy and enforce the reporting system.

### Tabulation and reporting

Focal points gather the duplicate death notification form and forward it to the coding unit. Causes of death are manually coded and underlying cause is selected using ICD-10 rules.^2^ Staff key the data into an electronic database (Oracle, Reedwood Shores, United States of America) developed in 2003 to facilitate storage, retrieval and analysis of mortality data. The database system includes basic logic checks and is linked to the national identification system at the Civil Status and Passports Department. The percentage of ill-defined conditions and unsuitable underlying causes are monitored to check quality and completeness. Additional training for coders and physicians is conducted on a regular basis. Data are organized by ICD chapter and three-digit ICD codes. The database system generates a publicly-available annual mortality statistics report.^3^

## Relevant changes

**Fig. 1:**
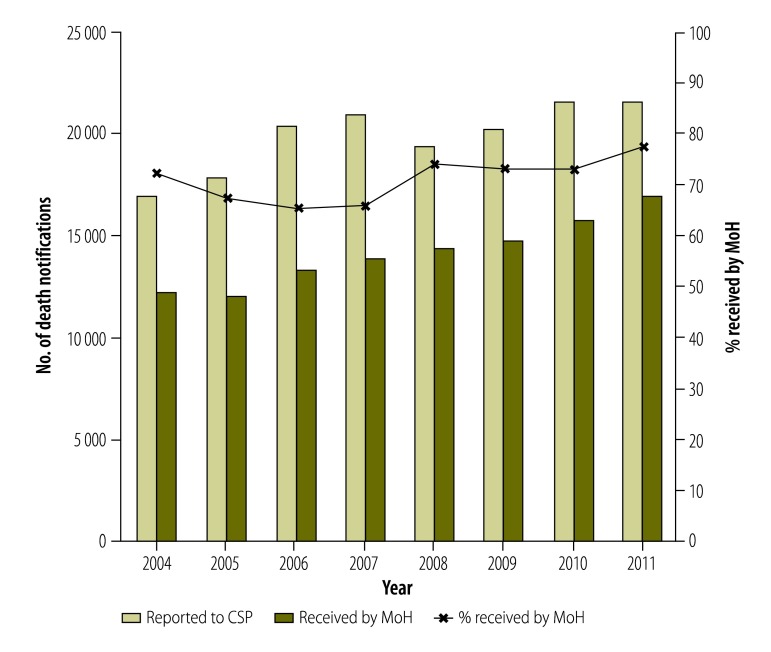
Death notifications, Jordan, 2004–2011

CSP: Civil Status and Passports Department; MoH: ministry of health.

The first data collected by the new mortality surveillance system (July to December 2003) showed consistency in the distribution of deaths by major cause as compared with the benchmark verbal autopsy study in 1995–1996.^1^ The ministry of health produced the first annual mortality statistics report in 2007, based on 2004 data.^4^ The report provided mortality statistics based on 71.9% of the death notification forms, a figure that increased to 77.7% by 2011 (Fig. 1). WHO recommends that less than 10% of deaths should be classified as caused by ill-defined conditions.^5^ By 2011, ill-defined conditions accounted for only 2.9% of deaths, similar to the USA (1.9%).^6^

A mortality statistics publication, tabulated by the ICD-10 classification, has been released annually since 2007, maintaining an interval approximately three years from the close of the data year to publication. Since 2008, mortality information has been submitted to WHO with four-digit coding, following the prescribed data template.^7^ In 2011, diseases of the circulatory system (ICD codes: I00-I99) remained the leading cause: 37.7% of deaths, (Shehab F and Walke H. Analysis of registered deaths in Jordan 1996–2000, unpublished observations, 2002), compared to 31.1% in the USA.^6^

A rapid assessment of the Jordanian civil registration and vital statistics system conducted in November 2012 documented improvement of the mortality surveillance system. Using a standard tool, Jordan scored in the highest category (87%, satisfactory), indicating that minor adjustments may be required in an otherwise well-functioning system (Table 1).^8^ Compliance with ICD practices, certification and coding methods were highlighted as strengths. Practices affecting the quality of cause-of-death data; coder qualification and training; quality of coding and data quality and plausibility checks were suggested as areas needing further improvement.

**Table 1:** Assessment of the civil registration and vital statistics system, Jordan, 2012

Function	Score (%)
Legal framework for civil registration and vital statistics	67
Registration infrastructure and resources	100
Organization and functioning of the vital statistics system	100
Completeness of registration of births and death	83
Data storage and transmission	100
ICD-compliant practices and certification within and outside hospitals	100
Practices affecting the quality of cause-of-death data	67
ICD coding practices	100
Coder qualification and training, and quality of coding	50
Data quality and plausibility checks	50
Data access, dissemination and use	92
Overall	87

ICD: International Statistical Classification of Diseases and Related Health Problems.

Note: The assessment was done using the *Rapid assessment of national civil registration and vital statistics systems*.^8^

## Lessons learnt

The coverage, completeness, timeliness and validity of the aggregated results of the Jordanian mortality surveillance system have greatly improved as a result of the project, but further improvements are possible. Implementation of the Iris system for automated coding is currently being considered to improve timeliness and quality.^9^ Continued improvement efforts would benefit from a formal assessment using an existing framework.^5^^,^^10^^,^^11^ The project has improved appreciation of the value of vital statistics. Data and reports from the mortality surveillance system are publicly available and are disseminated to all health directorates and other health agencies for planning purposes. This information has generated commitment from the government to achieve health-related Millennium Development Goals. Objectives in the ministry of health’s strategic plan are now informed by mortality data and various programmes have used the data to plan and measure improvement.^12^^,^^13^ The increased value placed on information from the mortality surveillance system has generated continued internal and external support for the system (Box 1).

Box 1 **Summary of main lessons learnt**

- An inter-agency collaboration, led by a mortality surveillance unit, was essential.
- The project did result in more complete and accurate death notification.
- The mortality reports generated have been useful for planning purposes.

Jordan, like other middle-income countries, is witnessing an epidemiological transition characterized by an increase in noncommunicable diseases. Mortality statistics are the principal means for assessing population-health status, identifying health problems, tracking progress, and comparing health status between countries. The ongoing effort to improve the mortality statistics in Jordan provides an example for other countries facing similar needs.
